# 

**Published:** 1951-04

**Authors:** 


### CONTENTS

| | Page |
|---|---|
| **Geriatrics.** I.—The Medical Problem of To-day: By C. T. Andrews, M.D., F.R.C.P. II.—Modern Developments: By T. S. Wilson, M.D. III.—The Local Problem: By William Hughes, M.D., M.R.C.P. | 35 |
| **Liver Function Tests.** By G. Kemp McGowan, B.A., B.M., B.Ch. | 46 |
| **Surgical Neurology.** By George Alexander, F.R.C.S. | 55 |
| **In Memoriam** | 58 |
| **Obituary: John Alexander Nixon** | 58 |
| **Meetings of Societies** | 60 |
| **Local Medical Notes** | 62 |

